# Evaluation and adaptation of a two-way text messaging intervention in the WIC breastfeeding peer counseling program: A qualitative analysis

**DOI:** 10.1371/journal.pone.0313779

**Published:** 2025-01-09

**Authors:** Josefa L. Martinez-Brockman, Josephine R. Granner, Brice Buchanan, Lisbette Acosta, Marilyn Lonczak, Lori Goeschel, Xiao Xu, Leslie Curry, Marcella Nunez-Smith, Rafael Pérez-Escamilla

**Affiliations:** 1 Department of General Internal Medicine, Yale University School of Medicine, New Haven, CT, United States of America; 2 Equity Research and Innovation Center, Department of General Internal Medicine, Yale University School of Medicine, New Haven, CT, United States of America; 3 West Haven VA Medical Center, West Haven, CT, United States of America; 4 State of Connecticut, Department of Public Health, Community, Family Health and Prevention Branch, Maternal Child Health and Access to Care Section, Special Supplemental Nutrition Program for Women, Infants and Children, Connecticut, United States of America; 5 Department of Obstetrics and Gynecology, Columbia University Vagelos College of Physicians and Surgeons, New York, NY, United States of America; 6 Department of Health Policy and Management, Yale University School of Public Health, New Haven, CT, United States of America; 7 Department of Social and Behavioral Sciences, Yale University School of Public Health, New Haven, CT, United States of America; Christiana Care/University of Delaware, UNITED STATES OF AMERICA

## Abstract

Breastfeeding (BF) is vital for maternal and infant health, yet post-hospital discharge support remains a challenge. The Special Supplemental Nutrition Program for Women, Infants, and Children (WIC) provides BF peer counseling prenatally and up to 1-year postpartum among low-income women in the United States. The Lactation Advice Through Texting Can Help (LATCH) intervention is an evidence-based two-way text messaging intervention that provides BF education and support in the WIC peer counseling program. The intervention is implemented by peer counselors (PCs) in the WIC program, with the supervision and support of a lactation consultant. The aim of this qualitative study was to assess the barriers and facilitators to the implementation of LATCH during the feasibility trial and to investigate strategies for adapting and scaling up the intervention. In-depth interviews with LATCH and PC program key informants aimed to evaluate the intervention and explore its adaptation and scale-up. Interviews were analyzed using line by line inductive thematic analysis. Findings were mapped to the Model for Adaptation Design and Impact (MADI) framework to describe the proposed adaptations, and to the Program Impact Pathways (PIP) framework to identify causal pathways and critical quality control points. Results demonstrate that LATCH facilitates continued engagement between PCs and WIC mothers; however, implementation feasibility issues remain. Suggested adaptations to LATCH include the use of an integrated comprehensive platform, ensuring continuity of care through an expanded spectrum of communication options, the need to develop a PC support model for “off hours” (non-business hours), and the need to obtain local WIC office management’s buy-in for the communications platform and the off-hours PC model. Critical quality control points were identified when results were mapped to the MADI and PIP frameworks. Implementing these changes has the potential to expand access to BF peer counseling support and improve BF equity among low-income women.

## Introduction

Breastfeeding (BF) is a complex biological and psychosocial dyadic behavior that improves the health of mothers and infants [1, 2] Lack of BF support after hospital discharge is a primary barrier to BF success. BF peer counseling is a promising community-based strategy to improve BF outcomes. Interventions that combine health systems and community-based services such as peer counseling have the largest effects on BF outcomes [3]. Low-cost, high-yield primary prevention interventions that address early life nutrition inequities, including BF outcomes, are essential to reducing preventable infant and maternal morbidity and mortality, improving child development, reducing healthcare costs, and achieving equity in BF outcomes [3].

The Special Supplemental Nutrition Program for Women, Infants, and Children (WIC) is a federal nutrition assistance program designed to safeguard the health and wellbeing of low-income pregnant and postpartum women, infants, and children up to 5 years of age at nutritional risk [4]. The program provides nutrition assessment, education and counseling, BF promotion and support, supplemental nutritious foods, and referrals to health and social service programs, to those at or below 185% of the federal poverty level. WIC currently reaches 51.2% of all eligible women, infants, and children [5]. A larger proportion of infants (78%of all eligible infants) receive WIC benefits, compared to women and children [5]. Breastfeeding education and support offered through the WIC program and the WIC Breastfeeding Peer Counseling (PC) Program is an integral part of the services provided, with the goal of increasing BF intensity, duration, and community support. Breastfeeding peer counselors (PCs) some of whom are former or current WIC participants with BF experience, support human lactation and encourage BF as the foundation of early life nutrition by providing basic BF information and support in a socially and culturally sensitive manner. They provide services under the supervision of an International Board-Certified Lactation Consultant (IBCLC; hereafter, lactation consultant (LC)) during the prenatal period and up to one-year postpartum. PCs are trained to consult with or refer complex BF questions and issues to the lactation consultant. While rates of BF initiation in the WIC program align with national averages, the rates of BF exclusivity and duration fall short of the objectives outlined in Healthy People 2030 [6]. The scale-up and sustainability of evidence-based PC interventions in the WIC population is an essential strategy to reduce BF disparities and maternal morbidity and mortality and improve child development [7].

### Lactation Advice Through Texting Can Help (LATCH)

The LATCH intervention was designed to complement the PC standard of care by providing WIC mothers and their PCs with two-way text messaging capabilities. LATCH is an evidence-based two-way text messaging intervention that provides BF education and support prenatally and up to 3 months postpartum. The intervention is implemented by PCs in the WIC program, with the supervision and support of a lactation consultant. LATCH was piloted (2011–2012) and later tested using a randomized controlled trial (RCT) design (2014–2016). The intervention and the text messaging schedule were co-designed with WIC PCs and lactation consultants, using the Health Action Process Approach (HAPA) model of behavior change as the theoretical framework [8, 9]. The text messaging schedule is based on the baby’s gestational age at enrollment and was designed so that the frequency and content of the behavior change messages changed during the prenatal, peripartum, and postpartum periods [10]. The results of the pilot and feasibility RCT are reported elsewhere [11–14]. The feasibility RCT established LATCH as a promising, theoretically sound, behavior change text messaging intervention that enhances and reinforces the WIC PC program [13]; however, questions remained about the scale-up, sustainability, and cost of the intervention.

### Purpose

The purpose of this qualitative implementation science study was to understand potential barriers and facilitators to the implementation, adaptation, and scale-up of the LATCH intervention. The specific aims were: (1) to evaluate barriers and facilitators to the implementation of the LATCH feasibility trial that occurred prior to the pandemic in the PC program (2014–2016); and (2) to explore how to adapt and scale-up the LATCH intervention in the PC program landscape transformed by the Covid-19 pandemic.

## Materials and methods

### Study design

This qualitative study used in-depth individual interviews with key partners which were analyzed using inductive thematic analysis. The study was approved by the Yale University Human Subjects Investigation Committee (protocol #: 2000033532; approved 09/14/2022) and the Connecticut Department of Public Health Human Investigations Committee (protocol #: 987; approved on 06/09/2023). Participants were required to provide written informed consent.

### Study setting, partners, and sample

This study was conducted in the state of CT in collaboration with the WIC PC Program. A brief history of the WIC PC program in CT is provided in the supplementary materials. In consultation with our CT WIC partners, purposive sampling was used to identify key partners, including WIC administrators, LCs, nutritionists, BF coordinators, and PCs, some of whom participated in the LATCH feasibility trial and some who did not. Saturation occurred when no new domains, themes or subthemes emerged—approximately mid-way through the coding exercise.

### Development of the in-depth interview guide

Four separate interview guides were developed, two for PC program administrators and PC program staff who participated in the LATCH feasibility trial, and two for those who did not participate in the LATCH study. The interview guides were developed by the study team, building on interview guides used in previous partner-engaged studies with BF peer counseling programs and the Model for Adaptation Design and Impact (MADI) framework [15]. Changes to the interview guides were made iteratively as the interviews proceeded to clarify questions and probe more effectively.

### Coding team and reflexivity statement

The four-member coding team drew from diverse backgrounds and areas of expertise. The coding team included 4 of the authors. Author 1, a Hispanic white female, study principal investigator, and faculty member in the school of medicine. Author 1 helped implement the LATCH feasibility trial and is a Certified Lactation Counselor with previous BF experience. Author 2 is a white female postdoctoral fellow in nursing and a doula. Author 3 is an African American male with a Master of Science in Public Health, and Author 4 is an Afro-Latina female undergraduate student with a background in psychology. The authors thoughts/biases were reflexively discussed as a group as they arose during the coding process. Before beginning the analysis, the team completed a qualitative coding training led by a qualitative methods expert.

### Application of the Model for Adaptation Design and Impact (MADI) and the Program Impact Pathways (PIP) frameworks

The overarching goal of this work is to apply the qualitative findings to the adaptation and scale-up of the LATCH intervention. With that in mind, the Model for Adaptation Design and Impact (MADI) framework was used to inform the development of the in-depth interview guides and to organize the results of the inductive thematic analysis around an intervention adaptation framework [15]. The MADI is an implementation science model designed to clearly document and understand adaptations made to existing evidence-based interventions and ultimately the impact of those adaptations on implementation and clinical outcomes. Importantly, the MADI was not used in the inductive thematic analysis to avoid prematurely imposing a preexisting conceptual structure on the data. Rather, it was applied to the results of the analysis to determine how the results mapped onto the model, describing the intervention adaptation process. The findings below are discussed in terms of the specific implementation outcomes they address, as described by Proctor and colleagues. Specifically, *acceptability* is described as “satisfaction with various aspects of the innovation (e.g., content, complexity, comfort, delivery, and credibility)”. *Adoption* refers to “uptake, utilization, initial implementation, or intention to try”. *Appropriateness* is “perceived fit, relevance, or compatibility”. *Feasibility* refers to “actual fit or utility, suitability for everyday use, or practicability” and *fidelity* refers to whether the intervention was “delivered as intended, adherence, integrity, quality of program delivery” [16]. Findings were applied to the MADI to describe the prospective intervention adaptation process. Each theme was also mapped to a relevant implementation outcome (Tables 1 and 2).

**Table 1:** Type and number of interviewees.

Role	Number
WIC Administrator*	2
WIC Nutritionist/IBCLC	3
WIC BF Coordinator	1
WIC Peer Counselor	6

*Each administrator completed two interviews

**Table 2:** Barriers and facilitators to the success of the LATCH feasibility trial (2014–2016).

Themes and Sub-Themes	Relevant IS Outcome(s)
**LATCH Facilitates Continued Engagement**	
Timely support and anticipatory guidance helps build trust and rapport between peer and mom	Acceptability
	Appropriateness
Timely and appropriate messages and message repetition leads to BF planning behaviors and BF self-efficacy	Acceptability
	Appropriateness
	Adoption
	Feasibility
**Feasibility Evidence**	
Interdisciplinary relationships and buy-in	Acceptability
WIC staff were co-designers of the intervention from the beginning	Appropriateness
LATCH led to changes in WIC PC standard of care, specifically using lessons learned from the RCT to train peers	Adoption
Reduces peer workload over time	Feasibility
Potential to aid in the retention of participants in the PC program	Sustainability
**Implementation Feasibility Issues**	
Need for continuous monitoring of the texting platform	Appropriateness
Timely responses	Feasibility
	Fidelity
Documentation requirements and associated language barriers	Appropriateness
	Feasibility
Automation issues (related to the automated text messaging schedule)	Appropriateness
Peer and IBCLC buy-in and variability in engagement	Adoption

The Program Impact Pathways (PIP) framework is designed to describe the necessary activities of a given intervention, and the relationships between activities that need to take place for an intervention to achieve its intended outcomes [17–19]. Different from a logic model, the PIP describes in a dynamic way the causal pathways across program activities, to reach the desired outcomes. An essential part of the PIP development process is to identify critical quality control points that can then be used to evaluate and monitor implementation progress, providing data informed feedback loops to intervention administrators and staff to sustain or improve program quality. The PIP models presented in this paper were established using the authors experience from the LATCH feasibility trial and the present qualitative findings. The PIP diagrams describe the activities, outcomes, and critical quality control points of the original LATCH model (implemented during the feasibility trial 2014–2016) and the adapted LATCH model identified through this qualitative research. The PIP models were then mapped to the Capability, Opportunity, Motivation and Behavior (COM-B) model of behavior change for designing and characterizing interventions and determining if the program is likely or not to have the “strength” to benefit its participants as intended [20].

### Documentation and evaluation approach

The principal investigator (Author 1) interviewed 12 key informants. Two of the 12 key informants were members of the WIC leadership team, and both had practical experience with the LATCH feasibility trial, as well as clinical and policy knowledge. Their interviews were more extensive, so we interviewed them twice, resulting in a total of 14 interviews. The first 7 interviews (5 key informants; 2 of whom completed 2 interviews each were conducted with WIC staff who participated in the LATCH pilot and feasibility trial and were used to address both research aims. These participants were provided with a 1-page overview of the study procedures one day prior to the interview to help refresh their memory about the LATCH trial. The other seven interviews were conducted with WIC staff who were not involved in the LATCH feasibility trial but were actively employed by WIC at the time of the interview and had experience providing PC services during the pandemic and infant formula shortage of 2022. All 14 interviews were used to address the second research aim. The interviews were audio and video recorded over Zoom and subsequently transcribed using a professional transcription service. Quality assurance checks were completed for each interview, comparing the transcript to the original video recording. The transcripts were analyzed using line-by-line inductive thematic analysis [21, 22]. This allowed for the themes to emerge naturally from the data rather than being imposed by pre-existing theoretical expectations.

The analytic procedure was based on Braun and Clarke’s (2006) six-step approach [21]. First, each team member individually read the transcript to familiarize themselves with the raw data. Second, coders independently generated and assigned initial codes to the data, identifying features of the data that appeared interesting or significant to the research questions. These codes were the basis the initial draft of the codebook. Third, the team met to discuss the initial codes, grouping them into potential themes. Fourth, themes were reviewed by the team after individually analyzing each transcript, engaging in robust line-by-line discussion of the transcripts. The codebook was iteratively refined during each discussion. Discrepancies were resolved through discussion until consensus was reached or a decision was postponed until additional transcripts were analyzed. Fifth, after repeating this procedure for all 14 transcripts, the themes were refined to ensure that the final codebook accurately represented the data and captured the range of perspectives shared by participants.

Final transcripts were imported into the qualitative data analysis software Dedoose 9.0 and audited for accuracy and appropriate application of codes, using the final version of the codebook developed in step 5. Questions and discrepancies were discussed and resolved through a negotiated coding approach with the coding team to improve reliability [23]. Results were exported from Dedoose into individual Word documents by code and synthesized using the MADI framework to produce the report (Braun & Clarke’s step 6).

## Results and discussion

### Interviews

A total of 14 interviews were conducted with 12 interviewees between December 2022 and June 2023. Five of the 12 interviewees were involved in the LATCH feasibility trial. Table 1 displays the role and number of interviewees in each role.

### Facilitators and barriers to the success of the LATCH feasibility trial (2014–2016)

The findings discussed below come from the first 7 interviews (5 participants) who were a part of the LATCH feasibility trial. Three major themes were identified: (a) LATCH facilitates continued engagement, (b) Feasibility evidence, and (c) Implementation feasibility issues. Themes and corresponding sub-themes (where applicable) are described below and presented in Table 2. Direct participant quotes are included to illustrate key themes and sub-themes. Any edits for clarity are identified in square brackets.

#### LATCH facilitates continued engagement

Two different sub-themes were identified and mapped to implementation outcomes. Interviewees noted that the LATCH intervention augmented the traditional methods of communication (phone and in-person visits) between peers and moms and demonstrated the following positive attributes: (1) timely support/anticipatory guidance helps build trust and rapport between peer and mom; and (2) timely and appropriate messages and message repetition leads to BF planning behaviors and self-efficacy.

First, the timely support and anticipatory guidance provided by the scheduled text messages *helped to build trust and rapport between peers and moms*. Enrolled moms received text messages according to their baby’s gestational age. As one supervising LC said, “What I like about LATCH, at the very least, they’re getting a piece of accurate information at the time when they probably need it, even though they don’t know that they need it". The automated messages helped to normalize the mothers’ experiences and establish close working relationships between peers and moms. One PC mentioned that the automated texts helped to establish a rapport with each new mom and opened new conversations:

It was something that helped us get closer to moms with those automatic texts that they would get. It was like they had that thing that say [*sic*], "Oh, she’s thinking about me. Oh, actually, this is the question that I have right now. This is what I really want to ask." Because sometimes they can forget about questions.—PC

Participants were asked to reach out to their PC once their baby was born. They did so by texting “BABY HERE”, or “BEBÉ AQUÍ” for Spanish speakers, when they went into labor or after the baby was born. One LC described the process as follows:

I think it built trust and rapport, which is …part of the benefit of peer services, very quickly. So, I think that, yes, some of those conversations would’ve happened [without automated texts]. What wouldn’t have happened so quickly is to inform the peer that the baby was born. And sometimes it really did make that connection between the peer and the participant more powerful because she would send a picture. So, she was really intimate with the peer very early.—WIC Administrator

This early notification of the baby’s birth (compared to the standard of care) was described by a WIC administrator as “the most critical part of LATCH…connecting with someone right away and right before delivery, knowing, keeping that on your forefront.”

Second, *timely and appropriate messages and message repetition led to BF planning behaviors and BF self-efficacy*. While these theoretical constructs were measured and mapped in our previous work [13], the BF planning process was described succinctly by one administrator:

I think it brought it to the forefront. Like, "I really do need to talk about it, or I really do need to tell my partner this is what I’m planning to do. Or I need to be able to go to the hospital and tell them, ’No, I’m breastfeeding my baby. Keep the baby in the room.‴ Because I don’t think *[hospital name redacted]* was Baby Friendly then. So, I think it really did—it added a lot more reinforcement to messages. And I think people need to hear things, see it, experience. They needed it all different ways. And we were only just communicating it verbally, but then they’re seeing it as well. And they could say to somebody, "Look, see, I got a message about it. I’m not kidding. This is healthy for my baby." So, it kind of gave them that reinforcement to share with others, if they wanted to.–WIC Administrator

The text message guidance also served to *build self-efficacy*. One LC shared:

So that part of it, I think, is extraordinary that those messages, they don’t take the place of the human voice or the human contact, but the mom can say, "I just read that what I’m going through is what all moms go through. Day three, I don’t have any milk." Every single woman who ever gave birth believes they don’t have milk on day three. Your body’s not doing what we think you’re supposed to be doing, but that is exactly what you’re supposed to be doing. You just—all those messages, "Remember, this will pass." …that’s part of what keeps you going. You need it.–Supervising LC

The messages reinforced and repeated messages that moms were hearing from their PC when they met with her in-person or over the phone. One PC described the smaller doses of information as highly beneficial for comprehension and retention:

"You’re 39 weeks at this time. Your breasts are getting ready. You already make [sic] colostrum. So excited, right?" So, you read that text, but it’s just a text that you read. It’s not like you’re getting a whole full thing, but it stays more in your mind when it’s a little information than when you are given a whole statement where you just don’t get everything from that statement. You just get a little bit of information, it stays longer in your brain.–PC

#### Feasibility evidence

Second, the interviewees brought to light pieces of evidence that support the *feasibility* of the LATCH intervention. They appreciated that the project was grounded in *interdisciplinary relationships and stakeholder buy-in*:

So, we had a physician on our side and really saw the power of what could be done at WIC and was so passionate about the funding that she had about this program and just really embraced [PC] and I. It was almost as important as the work that was being done… the connection we had with her and seeing her dedication as a physician was so refreshing.–WIC Administrator

WIC staff were *co-designers of the intervention* from the very beginning of the pilot project. They appreciated that their feedback was taken seriously and used to improve the content and timing of the text messages. One supervising LC said, “And when we made the messaging, it was really based on kind of real-life experience, real life of how best to communicate with the moms, motivational interviewing, and using our platform of how we talk to the moms.”

The two-way text messaging platform allowed for a *reduction in the peer workload over time* because some of the education and support was automated, so the PC did not have to generate this information themselves. It also allowed the peer and her supervising LC to work more seamlessly together. According to one LC:

It gave our peer counselor another tool and a more effective tool to communicate. And I was able to take a second to jump on those two-way, just to oversee and see what kind of conversations were going on. And if everything was smooth, we didn’t need to talk to each other face-to-face. We could effectively assess a nursing dyad and we didn’t need to always directly meet face-to-face. So, it’s always going to make life easier when you have that kind of technology and that kind of step-up of care that that platform represented.–Supervising LC

LATCH also *led to changes in the PC program standard of care*. Specifically, lessons learned by the LCs and peers are currently used to train new peers in best practices for text message communication.

And I’ve actually used examples of LATCH when I’m training peers now, and I’ll say to them, "When we were part of a study, we would do a lot of texting, but then there were times where we would need to call the participant." And I said, "And that might need to happen, and you might have a mom who doesn’t want to talk to you. But if that’s the case, you can just say, ’Five minutes. That’s all I need. Two minutes. That’s all I need. I just want to clarify. I just want to ask a few questions, and then we can go right back to texting.‴–WIC Administrator

Finally, WIC administrators saw LATCH as a tool with the potential to aid in the retention of participants in the PC program. Referring to the automated messages and the two-way texting platform, one administrator said:

So having that [the automated text messages] as something that’s just happening is a way to keep connected, which is what we need…We don’t want people to fall through the cracks because, as it is now, people are having their benefits issued and then their next appointment is in between one and three months. Lots of times, three months. A lot of things happen in that three-month period. So hopefully we’ll reduce the amount of people that are coming back in three months and saying, "I stopped breastfeeding," or "I’m reducing breastfeeding," or "I’m thinking of stopping breastfeeding." If there’s more points that they potentially are hearing from WIC, regardless if it’s human or AI or whatever, it’s still the ability to reach out with relevant information.–WIC Administrator

#### Implementation feasibility issues

Four primary sub-themes were identified and mapped to relevant implementation outcomes (Table 2). Implementation feasibility issues included: (1) the need for continuous monitoring of the two-way texting platform; (2) the documentation required of the PCs and associated language barriers; (3) issues that arose due to the automated nature of the text messaging schedule; and (4) peer buy-in and variability. The research team collaborated with the peer teams to monitor the platform for incoming text messages. The PCs and their supervising LCs had to learn how to monitor the two-way texting platform independently so they could respond to each participant in a timely manner. While they appreciated the ability to monitor conversations and address BF issues in real time, *the need for continuous monitoring of the platform* added to their workload:

So, I liked it. As a person who was overseeing it in two places, it’s a little—it was cumbersome for the person. There’s no way I could monitor all that dialogue all the time. It was a lot. It was a lot, a lot, a lot. I didn’t ignore it. I did some of it every day, but it could be a full-time thing for that person monitoring. And you could really see a lot of what was happening [between PCs and moms].–Supervising LC

The *need for regular monitoring* was compounded by the fact that most PCs worked part-time and thus monitoring of the platform was necessarily divided between the PCs, their supervising LC, and research staff. While the frequency and content of the text messages was deemed acceptable, *timely responses* to the incoming text messages were considered essential:

…because we knew the date of birth. So, if we knew it was day two and there was one meconium stool and that was on Friday afternoon, then we’re not really responding to that until we logged [in] on Monday. And that could either resolve very quickly and be fine or be a disaster by Monday. So, I never felt like participants were getting too many messages. I was more worried about the peers getting back to the participant quickly with the right information and the right support.–WIC Administrator

Second, the *documentation that was required* of the WIC staff during the feasibility trial, beyond their existing documentation responsibilities, was seen as a barrier. Staff were asked to document each instance where a conversation moved from the two-way text messaging platform to a phone call, or an in-person visit. This record keeping was done so researchers could follow-up on the outcome of the phone call to determine whether the BF (or other) issue was resolved. This created an additional documentation step for the PCs. One WIC administrator described it as follows:

But I think for the most part, potentially the barrier would be documenting a text exchange. So, say an automated text goes out at some point in time that’s off the [WIC] service schedule, and then the peer has to engage with the participant based on questions. That would need to be documented. So, there’s a burden on documentation, but the whole point is to have communication with participants about their questions…and improving duration of breastfeeding. So, yes, I think its what people perceive as their job and if you’re given more work. But I feel like most of the peers want their participants to be breastfeeding as long as possible. So, if there is anything that will help facilitate that, I think that would make sense to them.–WIC Administrator

Although the text messaging intervention was offered in English and in Spanish, the text messaging platform and the documentation required during the study may have been burdensome for PCs whose first language was not English. Addressing the feasibility and appropriateness of LATCH for *peers whose first language was not English* will be important in scaling-up the intervention. Issues also arose related to the *automated nature* of the text messaging schedule and the need for continuous monitoring of the texting platform. Messages were sent out on a predetermined schedule based on the baby’s due date. One supervising LC identified the need to improve the automation features:

As I recall, the frequency and content were spot on. I think we had one sort of glitchy situation where one of our participants miscarried and the [automated] text messaging came out to her. And I don’t know if it was something on our part, that we didn’t take her out soon enough of the pool, or if she didn’t let us know until she got a text and then—that kind of thing. So, like anything, you have those where you work out the bugs.–Supervising LC

The final implementation issue was *getting PC and LC buy-in for the text messaging platform*, *and variability in buy-in*. During the feasibility RCT, some PCs were more engaged with the intervention and the text messaging platform than others. The interviewees noted that engagement with LATCH was very person-dependent in that everyone is different in how they like to communicate. To improve engagement and uptake, PCs and LCs will need to be trained and educated about the text messaging schedule, the utility of the two-way text messaging platform, and how it is designed to improve communication and continuity of care:

I think that’s probably peer dependent…because I know that there was some differences based on the site, right? So, I think some sites probably had a better understanding of how effective texting could be and how that could help them with their caseload. Where I feel like some of the other sites might have seen them [the texts and messaging platform] as burdensome. I don’t think it’s going to be viewed moving forward as burdensome because this is how this generation communicates…So I think it all depends on the individual. Because again, if it’s part of the job, if the job evolves to have better documentation…I mean, the whole point is to have continuity of care.–WIC Administrator

### Suggested adaptations and implementation strategies to facilitate the integration and scaling-up of LATCH

The second research question of this study aimed to identify how to adapt and scale-up the LATCH intervention in the post-pandemic WIC landscape. Two major themes were identified: (a) suggested adaptations, and (b) scaling-up concerns (Table 3). When the concern about scale-up and the suggested adaptation related closely, the sub-themes are presented together. LATCH operated through a two-way text messaging platform and partners suggested how to expand the functionality of the platform to better serve the needs of the peers and moms. First, key partners expressed the need for an *integrated comprehensive platform*. During the feasibility RCT, most PCs texted participants from their computer’s web browser but current peers expressed the desire for computer access and an app on their phone for greater flexibility. Peers highlighted the need for a user-friendly interface that allows them to respond via computer or smartphone, making the platform more accessible and convenient:

The process itself is tedious. It’s one more website. So, I’m thinking if it’s an app that’s already on your work phone, where both mom and the peer can have access to it, even if it’s like—it still presents as the same as the website, but easier access. For instance, you just log in with the code on your phone, and it’s instant. Even like if a mom texts, it will show”–PC

**Table 3:** Suggested adaptations and implementation strategies to facilitate the integration and scale-up of LATCH.

Themes and Sub-Themes		
Suggested Adaptations	Scaling-up Concerns	Relevant IS Outcome(s)
**Integrated Comprehensive Platform and Off-Hours PC Model**		
	Integration into existing service schedule	Feasibility
	Managing the monitoring of the platform off-hours	Appropriateness
	Ensuring appropriate staffing	
**Ensuring Continuity of Care Through an Expanded Spectrum of Communication Options**		
System generated notifications	Managing the monitoring of the platform off-hours	Feasibility
Clear emergency/urgent protocol		
	Ensuring continuity of care	
	Clearly defined boundaries for peers to set with moms regarding their hours of availability	
Live availability	Assess whether the participant is meeting their goals	Appropriateness
Ability to ask open-ended questions		
		Feasibility
Modalities for peer to mom communication	n/a	Appropriateness
		Feasibility
Translation services	n/a	Appropriateness
**Local WIC Office Management’s Buy-in to the 2-way Platform and Off-Hours Coverage**		
	n/a	Feasibility
		Adoption

This could also reduce the difficulty of documenting the text message conversations with mothers, if they could be automatically integrated into participant records. As one PC suggested: “And that platform would be nice to have everything all put together…because right now we’re looking at our agendas, on our WIC platform, sticky notes if we put it on our computer… So, it’d be nice to just have it all organized in that one platform.” This would also facilitate LATCH *integration into the existing service schedule*, another commonly raised scaling up concern:

So, if we merge LATCH into the service schedule with appropriate communication—not just it’s sent out and who knows and it counts. There needs to be dialogue. We need to know that the participant is meeting the goals that they have set for themselves. Then I think that would help our program because we would be able to "count" those contacts as part of the service schedule.–WIC Administrator

Most partners believed this system could be beneficial and raised the scaling up concern of *ensuring appropriate staffing for the off-hours platform*. Peers said they would be willing to work in an off-hours (non-business hours) model, and would appreciate the added flexibility:

That’s an amazing, amazing idea for both moms and the PC. People want flexibility. Flexibility of location where to work and flexibility of schedules. For instance… you have children drop off, children pick up. If you can work around that time, that gives you flexibility. You can work from home, that gives you flexibility. You go to the office, again, people prefer flexibility. So that accommodates so many different people’s schedule and lifestyles and dynamics. But also offer those after-hours, like you mentioned. There are not business hours, where babies are like, "Well, after 5:00 p.m. I’m okay…" [Laughter].—PC

One stakeholder raised the idea of a flat rate for PCs who are on call. The flat rate would ensure that a PC is always available to answer participant’s questions. There is also a need for an on-call supervisor (a LC), with PCs and LCs compensated on a per-call basis for work done during on-call hours. The ability for PCs to yield to an on-call LC during off-hours coverage follows the current standard of care.

“I feel like there needs to be some sort of streamline on-call manager. And is that an IBCLC? I think it’s almost like you need to have it be a peer, but then you need to have an IBCLC sort of on call. And …you’re paying them well… for the hours they work. Maybe you give a stipend of $30 per shift just to be on call, and then you pay per time you reach out to either party. But I do think that for all intents and purposes … if it’s immediately outside of the peer scope, then they immediately call the IBCLC. But there’s a lot that probably could be covered by the peer.”–WIC Administrator

Second, partners discussed *continuity of care through an expanded spectrum of communication options* to better support mothers. Options included: system generated notifications, providing live availability of PCs on the LATCH platform in the context of an off-hours peer support model, training peers to ask open-ended questions on the platform, offering different modalities for peer to mom communication, and using translation services. Both BF mothers and PCs desire flexibility and evenings and weekend are when many mothers have the time to call their PC. In reference to the off-hours support model one PC said,

“The fact is that we’re serving a community that has lots of stuff going on… These moms might not really sit down and think about what is going well and what’s not going well until 11 o’clock at night. And that might be when they have a little bit of clarity and could talk. And if that’s when they could talk, it would be nice to be able to meet them where they’re at.”—Supervising LC

Continuity of care was an important cross-cutting theme in discussions of suggested adaptations with respect to internal WIC communications. Partners were adamant that the off-hours support model would need to ensure continuity of care between the on-call staff and regular WIC staff. One suggested adaptation was *system-generated notifications* to improve internal communication that would facilitate follow-up of participant’s concerns and BF care coordination. Specifically, it is crucial to close the communication loop by notifying the WIC office and the participants’ regular PC about any calls received during off-hours:

So, to end the loop with a way that there is a system-generated [notification] that’s coming via a clinic, and then the clinic is getting notification of who called and received guidance… there’s interchange within our CT WIC system. So, the peer is documenting in the CT WIC system. But unless somebody’s going into their chart, they’re not going to know there’s a note in there. It’s not like the thing flashes and says there’s been activity here. So, we need to have some sort of checks and balances so that they’re calling in a pinch, but yet we’re following up to make sure whatever was going on is resolved. -WIC Administrator

Along the same line, interviewees identified the need to *establish a clear emergency/urgent response* protocol, whether the off-hours model was implemented or not. When automated texts are sent to participants, it is critical to communicate whether someone is there to read their response. One WIC administrator asked, “If a participant reaches out to a peer and it’s an emergency, and its off hours… how can we push a notification that’s like, "If this is an emergency, call your healthcare provider"?” This type of mechanism may increase safety for mothers and infants and protect WIC from liability.

Similarly, partners emphasized the need for clear communication of peer availability with mothers. Especially considering the suggested off-hours PC support model, partners raised the scaling up concern of *clearly defined boundaries for peers to set with participants for their regular versus on-call hours*:

When our peers sit with the clients and they’re establishing that rapport, it’s setting those gentle boundaries. So, if they’re not on-call 24/7, letting that client know that. "Well, I’m here to support you, but again, it’s sort of—this is where my scope goes to. This is my boundary, and I really cannot cross that boundary."–Supervising LC

Additionally, training peers to *ask open-ended questions* on the LATCH platform will allow for a more personalized approach that builds trust, rapport, and provides targeted information based on what the participant truly needs help with. Therefore, a scaling up concern was how to *accurately assess whether a participant is meeting their individual goals*. Designing that metric is critical if LATCH is to be integrated into the existing service schedule.

Peers also reported variability in mothers’ preferred *modality of communication* and suggested offering different modalities of PC to mom communication. Some moms prefer video conferencing, while others might not want to be on camera, and others might want an in-person visit. Including different communication/interaction options in the LATCH platform would allow PCs to individualize care to better meet mothers’ needs. In addition, some PCs and many mothers have a first language that is not English, so integrating *translation services* will also improve communication.

Finally, a necessary ingredient identified for the success of scaling up LATCH was *local WIC office management’s buy-in to the two-way texting platform and off-hours support model*. During the previous LATCH project, local WIC leaders encouraged a high degree of agency, professional trust, and support for those implementing: “[They said], "Here’s the ball. Run with it” according to one supervising LC. When scaling up the LATCH program, support and full backing from local WIC administrators will be even more important. There is a need for ongoing conversations with state-level directors and local administrators about their commitment to supporting and sustaining LATCH:

Yes, the peer and the IBCLC might be very willing, very passionate, very excited about this. We’re on board, we’re there. But then if we don’t have that 100% support from the management above us, yeah, that could get sticky. I think that would be a conversation to have with the state level and the directors of the programs, to say, "Where does your commitment lie for your program, so that if the peer and the IBCLC are fielding a call at 2:00 am, if their start time was 8:00 am, will you give them till 9:00 am? Will you give them that extra hour? Will that be something that would work?" And I can already hear in my mind all the naysayers. "Well, we have schedules and client services, and the state says we need to do this and that."—Supervising LC

#### Application of the MADI and PIP frameworks

The results presented above were applied to the MADI framework such that themes and subthemes were mapped to the following implementation outcomes: adoption, feasibility, appropriateness, acceptability, fidelity, and sustainability of LATCH, from the perspective of those who implemented it during the feasibility trial (Tables 2 and 3). Other partners discussed the intervention from the perspective of those who would implement it in the future in a WIC landscape that has moved to virtual service provision due to the pandemic. Key partners concluded that the current WIC landscape is indeed well primed for the integration and scale up of LATCH. The MADI framework for the off-hours PC model (S1 Fig.) and the Logic Model (S1 Table) are presented in the supplementary materials.

**Fig 1:**
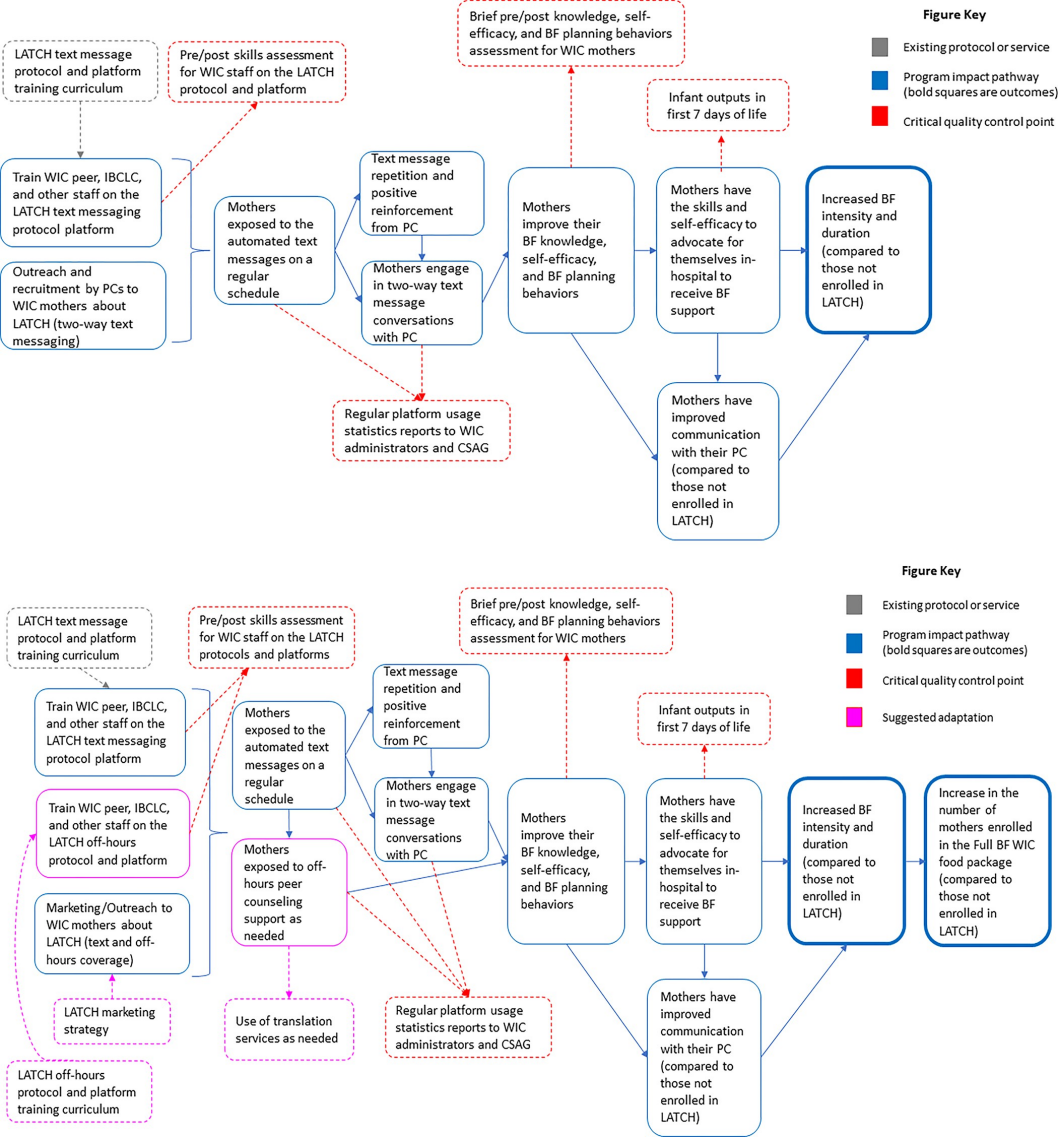
Original and adapted LATCH PIP models. In the depicted PIP models, existing protocols or services are those that were established prior to the feasibility trial (Fig 1A, gray boxes). Protocols or services that will need to be established during the co-development phase of the off-hours PC model are shown in Fig 1B (pink boxes) as suggested adaptations. Pathways from program activities to program outcomes are shown in blue. Critical quality control points are shown in red. **a.** LATCH Feasibility Trial Program Impact Pathways Framework in the WIC Program (2014–2016). **b.** Adapted LATCH Program Impact Pathways Framework with Off-hours PC Support in the WIC Program.

The PIP model for the original LATCH intervention (Fig 1A) and the necessary adaptations to support the off-hours PC model (Fig 1B) are presented below. In the original model (Fig 1A), WIC peers, LCs, and administrators were trained on the LATCH protocol and outreach to WIC mothers was conducted by PCs. Mothers enrolled in the intervention arm of the study were exposed to the automated text messages on a predetermined schedule based on the baby’s gestational age. Text message repetition led to positive reinforcement of the BF messages and mothers were encouraged to engage in two-way text message conversations with their PC. In turn, they improved their BF knowledge, self-efficacy, and BF planning behaviors. Mothers also improved communication with their PC and developed the skills and self-efficacy to advocate for themselves in the hospital during and after giving birth. These activities were designed to lead to increased BF intensity and duration compared to those not enrolled in LATCH. In the adapted LATCH framework (Fig 1B) the new components of the off hours PC model have been added as well as a new outcome measuring enrollment in WIC food packages. Critical quality control points represent data collection points that are essential to the ongoing evaluation and monitoring of the intervention. They will be monitored using WIC PC program administrative data for indicators that are Specific, Measurable, Achievable, Relevant, and Time-bound (SMART) and sustainable.

#### Mapping of PIP to COM-B behavior change model

The detailed mapping of each PIP diagram to the COM-B model can be found in S2 Table. The purpose of this mapping exercise was to understand whether the suggested adaptation of the off-hours PC model could be reasonably expected to lead to increases in BF intensity and duration once the intervention is scaled up. Intervention activities found in each PIP framework were mapped to one or more components of the COM-B (capability, opportunity, motivation, and/or behavior) model and their respective sub-classifications, for example psychological capability. As S2 Table shows, the intervention activities in the enhanced LATCH model (that included off hours PC support) map to all six subclassifications of the COM-B model, indicating that the adapted LATCH model includes the individual, social, and physical/contextual conditions that need to be in place to support WIC mother’s BF goals.

## Discussion

This study described the barriers and facilitators to the implementation of the LATCH feasibility trial and described ideas from key partners to adapt and scale-up the LATCH intervention. Findings revealed that the process of co-designing the LATCH intervention created a high degree of acceptability across all levels of PC program staff and that LATCH led to changes in the PC program standard of care. Partners involved in the feasibility trial noted that LATCH facilitates continued engagement, builds trust and rapport, and leads to BF planning behaviors and BF self-efficacy. However, important implementation issues remain including creating an integrated comprehensive communications platform, focusing on continuity of BF care through an expanded spectrum of communication options, and obtaining local WIC office management buy-in.

An integrated comprehensive communications platform will ensure that LATCH is implemented with fidelity, is feasible, versatile, and flexible, and suitable for everyday use. Comprehensive training on the integrated platform will ensure adoption and uptake of LATCH and coordinated integration into the standard of care. An ideal platform would incorporate two-way text messaging, automated text messaging, and a phone line for off-hours support. The staffing and support protocols for the off-hours support model need to be co-designed with WIC administrators, staff, and participants. Second, there must be a strong focus on continuity of BF care through an expanded spectrum of communication options. Providing additional avenues through which participants can contact their PC will help to ensure they reach out when they need help. An integrated platform would also improve internal communications between WIC staff by automating some communication procedures, which will ensure continuity of care. Advances in the technology of the text messaging platforms will facilitate the closure of the continuity of care loop. One example is the ability to use an Application Programing Interfaces (APIs) to integrate with other platforms. Finally, a concerted effort must be made to obtain local WIC office management’s buy-in for integration of LATCH into the standard of care. These design issues will be addressed in the next phase of LATCH where researchers will co-design the off-hours support model with key partners at the state and local levels. Partners believe that integrating LATCH into the standard of care will greatly enhance the support they can provide to BF mothers in the WIC program.

Other studies have used co-design methodologies to develop and implement breastfeeding interventions in the US and globally. In fact, co-design methods for intervention development and evaluation are acceptable and feasible in mHealth research like LATCH, and when working with historically marginalized communities, including low-income communities [24–26]. In CT, the Breastfeeding Heritage and Pride (BHP) program provides low-income women with BF peer counseling and referrals to social services [27, 28]. The BHP program began more than 20 years ago using a community-based participatory approach in the formative phase of intervention development and has continued to apply this approach to the implementation, monitoring and evaluation, and adaptation of the program [29, 30]. Qualitative methods are an essential component of the co-design process. For example, in-depth interviews with key partners allowed researchers to identify and address BF barriers specific to the Latino community during the BHP design phase.

A study in South Africa used co-design methods to develop an app to motivate mothers to donate surplus breast milk to their local milk bank. Specifically, they employed brainstorming sessions, workshops, surveys, follow-up interviews, observations, and cultural probes with members of their target audience in the app development phase. In Ethiopia, researchers used a co-design approach to develop and implement an mHealth intervention targeting fathers and mothers to improve breastfeeding behavior [31]. Parents were involved in the development, pretesting, and piloting of the text messages about infant feeding. This led to 97% of parents reading the text messages and 90% wanted to continue to receive text messages in the future [31]. And finally, the LATCH pilot study used focus groups and surveys with WIC mothers to assess the acceptability of the text message timing, content, and dose [11]. Co-design methodologies are varied and must be selected to meet the needs of the target population and research project. The evidence strongly supports the use of co-design methodologies in the development, implementation, evaluation, and monitoring of breastfeeding interventions targeting low-income women and their families. The co-design process will ensure that the intervention is appropriate, acceptable, and feasible to the target population.

The combined application and integration of the MADI, PIP, and COM-B frameworks demonstrate that LATCH is an intervention that can be adapted to suit the needs of WIC administrators, staff, and participants. Applying the MADI (S1 Fig.) allowed for a clear description of the need for the off-hours support model as detailed by key partners and ultimately the impact of the adaptation on implementation and clinical outcomes. The PIP analyses (Fig 1) described the causal pathways between intervention activities and identified critical quality control points for monitoring and evaluation purposes. The mapping to the COM-B framework (S2 Table) demonstrated the robust nature of LATCH in that it has the potential to provide the individual, physical, and social conditions needed to successfully support WIC mother’s BF goals. Taken together, these three frameworks demonstrate the nature of the adaptation (MADI), how the adaptation maps to the original intervention (PIP), and whether the adapted version of LATCH has the “strength” to benefit participants as intended (COM-B).

The evidence-based LATCH intervention was recently included in a systematic review and Meta-Analysis that found that text messaging interventions conducted weekly during both the prenatal and postpartum periods are most effective in improving rates of exclusive BF [32]. Additionally, the American Academy of Breastfeeding Medicine’s clinical protocol for discharge of the maternal-infant dyad after hospital birth identified LATCH as an educational intervention that can be used to support maternal BF self-efficacy, proper BF mechanics, connections to health care providers, and BF protection, promotion and support more broadly [33]. The present qualitative analysis adds to the body of evidence supporting the integration of LATCH into the WIC PC standard of care. It also supports the use of a collaborative user-centered approach to intervention development and adaptation centered around program co-design and evaluation. This process has allowed us to build a coalition of WIC administrators, staff, scientific advisors, and WIC mothers that oversee intervention design, implementation, and evaluation procedures in CT. In-depth interviews allowed researchers to understand how to adapt LATCH to better serve the needs of the WIC population and WIC staff and understand the facilitators and barriers to integration of LATCH. Another strength of this study was the coding team, who lent their diverse backgrounds and varied perspectives to the data analysis process. The primary study limitation is that these interviews began 6 years after the feasibility RCT ended, and the experiences that participants shared may have been influenced by recall bias. However, respondents involved in the feasibility RCT did not have difficulty recalling specific details of the implementation experience. It is possible however, that the staff involved in the LATCH feasibility trial, who remained at WIC and were able to be interviewed for this study, had a more positive experience with the intervention than those staff who left WIC and who were unable to be contacted for an interview. Additionally, the present study focused only on the experiences of the WIC administrators and staff who implemented the intervention and did not include WIC mothers who participated in the intervention. A separate series of interviews were conducted with current WIC mothers to understand their communication needs and obtain feedback on the proposed off-hours BF support model (results to be published separately).

## Conclusions

Two separate U.S. Surgeon General reports identified BF peer counseling in the WIC program as an essential service to support low-income women to achieve their BF goals and reduce disparities in BF outcomes [34, 35]. More recently, the *Biden-Harris Administration National Strategy on Hunger*, *Nutrition*, *and Health* recommended the expansion of BF counseling nation-wide [36]. Evidence-based BF peer counseling interventions are essential to the expansion of BF support in the US. The LATCH intervention is one such intervention that will expand access to BF support among mothers enrolled in the WIC PC program. Using an implementation science approach for the co-design, implementation, and evaluation of LATCH will help disseminate LATCH with off-hours BF support to other WIC settings outside of CT and understand the adaptations that may be needed to function efficiently across different contexts.

## Supporting information

S1 Appendix A brief history of the special supplemental nutrition program for Women, Infants, and Children (WIC) breastfeeding peer counseling program in CT. (DOCX)

S1 Fig The off-hours PC model applied to the MADI. (DOCX)

S1 Table LATCH logic model, including the off-hours PC model*. (DOCX)

S2 Table Mapping the program impact pathways framework to the Capability, Opportunity, Motivation and Behavior (COM-B) system for characterizing and designing behavior change interventions*. (DOCX)
