# Supplementary material for: Evaluation and adaptation of a two-way text messaging intervention in the WIC breastfeeding peer counseling program: A qualitative analysis
Source: PLoS One. 2025 Jan 9;20(1):e0313779. doi: 10.1371/journal.pone.0313779 (PMC11717301; doi:10.1371/journal.pone.0313779)
Supplement: S1 Appendix — (DOCX) [file pone.0313779.s001.docx]

**Supplementary Materials**

**S1 Appendix. A Brief History of the Special Supplemental Nutrition Program for Women, Infants, and Children (WIC) Breastfeeding Peer Counseling Program in CT**

In Connecticut (CT), the Department of Public Health administers the Special Supplemental Nutrition Program for Women, Infants, and Children (WIC) Program via nine local agency contractors which operate 22 permanent sites. Breastfeeding peer counseling in the WIC program began 20 years ago. Nationwide in 2004, approximately $15 million in federal funding became available to support the WIC PC program. Funding for the PC program is allocated to state agencies based on the number of prenatal and breastfeeding WIC participants. These PC funds can only be used to support implementation or expansion of existing WIC peer counseling services. In CT, from 2004-2010, the WIC PC funds supported efforts of the Hispanic Health Council’s Breastfeeding Heritage and Pride Program (BHP) in the Hartford area (Hartford Hospital) and expanded to New Haven (Yale New Haven Hospital) because the BHP model met the required components for PC set forth by the United States Department of Agriculture’s Food and Nutrition Service [1, 2]. Thus, PC was provided to WIC participants served by the Hartford and New Haven local WIC agencies. Nationally, in 2010 the WIC PC program funding increased to $80 million. The CT WIC program was allocated approximately $500,000 in WIC PC funds. Those funds supported both BHP programs and allowed the addition of three WIC clinic-based PC programs at its local agency contractors, all of which employed nutritionists certified as International Board-Certified Lactation Consultants to mentor PCs. Today, the CT WIC PC program is available in 8 of its 9 local agencies and at 12 local agency sites (some local agencies have more than one site where PC is offered). Statewide in CT in FY2022, 11,356 infants were served by the WIC program. Of those, 1,181 were fully breastfed (10.4%), and 3,286 were partially breastfed (28.9%) for a total of 4,467 breastfed infants (39.4%) [3]. Approximately, 1,015 dyads were enrolled in the PC program in FY2023.

References

[1] D. J. Chapman, G. Damio, S. Young, and R. Perez-Escamilla, "Effectiveness of breastfeeding peer counseling in a low-income, predominantly Latina population: a randomized controlled trial," (in English), *Arch Pediatr Adolesc Med,* vol. 158, no. 9, pp. 897-902, Sep 2004.

[2] A. K. Anderson, G. Damio, S. Young, D. J. Chapman, and R. Perez-Escamilla, "A randomized trial assessing the efficacy of peer counseling on exclusive breastfeeding in a predominantly Latina low-income community," (in English), *Arch Pediatr Adolesc Med,* vol. 159, no. 9, pp. 836-41, Sep 2005.

[3] United States Department of Agriculture (USDA), "Fiscal Year 2022 WIC Breastfeeding Data Local Agency Report," 2022. [Online]. Available: chrome-extension://efaidnbmnnnibpcajpcglclefindmkaj/https://fns-prod.azureedge.us/sites/default/files/resource-files/FY2022-BFDLA-Report.pdf
