# Supplementary material for: Evaluation and adaptation of a two-way text messaging intervention in the WIC breastfeeding peer counseling program: A qualitative analysis
Source: PLoS One. 2025 Jan 9;20(1):e0313779. doi: 10.1371/journal.pone.0313779 (PMC11717301; doi:10.1371/journal.pone.0313779)
Supplement: S1 Fig — (DOCX) [file pone.0313779.s002.docx]

**Supplementary Materials**

**Application of the MADI Framework to Findings**


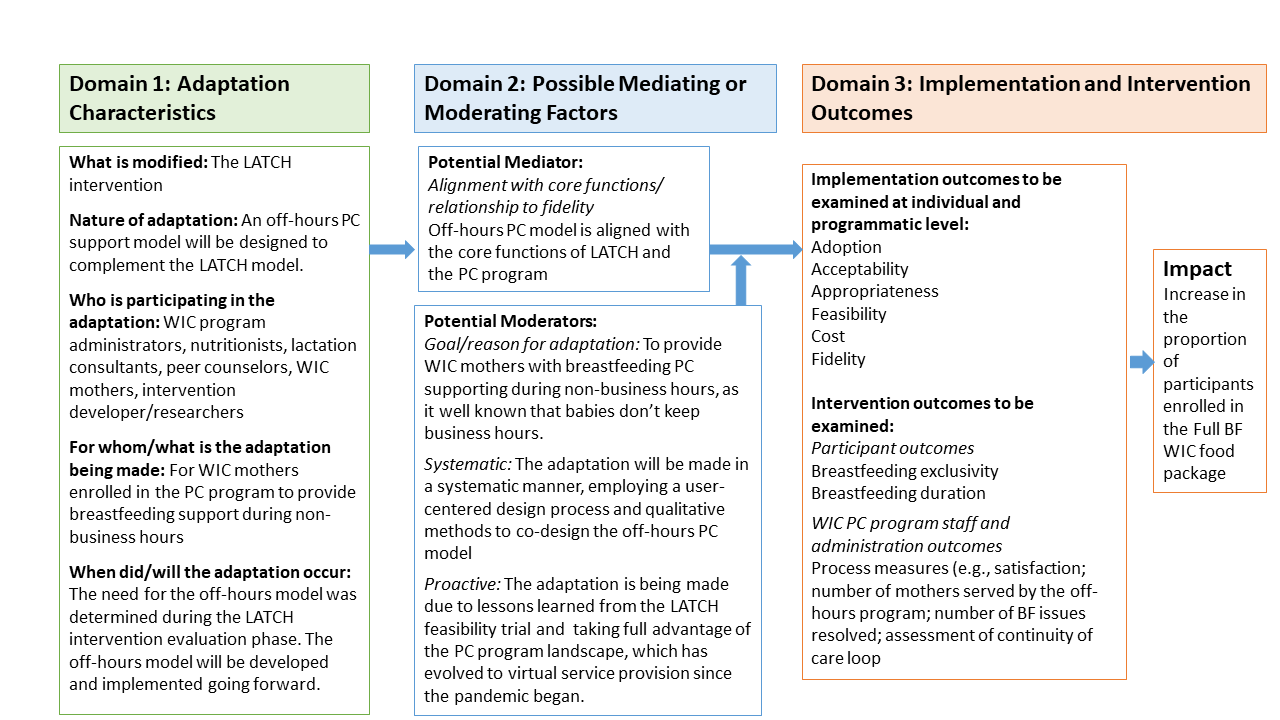
Key partners determined that a non-business hours breastfeeding peer counseling support model is needed as an adjunct to the LATCH model in the WIC PC program. Figure 1 below displays the use of the MADI to detail the need for the off-hours support model.

**S1 Figure. The off-hours PC model applied to the MADI framework**
