# Supplementary material for: Evaluation and adaptation of a two-way text messaging intervention in the WIC breastfeeding peer counseling program: A qualitative analysis
Source: PLoS One. 2025 Jan 9;20(1):e0313779. doi: 10.1371/journal.pone.0313779 (PMC11717301; doi:10.1371/journal.pone.0313779)
Supplement: S1 Table — (DOCX) [file pone.0313779.s003.docx]

**Supplementary Materials**

**S1 Table. LATCH logic model, including the off-hours PC model***

| **Assumptions** | **Beneficiaries** | **Inputs** | **Activities** | **Target Population** | **Products/Outputs** (short term goals) | **Outcomes** (medium term goals;  changes in capacity and behaviors) | **Impacts**  (long term goals; changes in health and well-being) |
| --- | --- | --- | --- | --- | --- | --- | --- |
| Trained WIC personnel using the Designated Breastfeeding Expert (DBE) curriculum  Existing policies and practices for yielding difficult BF issues from PC to LC  Existing emergency protocols for the WIC PC program  **LATCH standard operating procedures manual**  **LATCH training manual**  **Off-Hours support model - standard operating procedures manual**  **Off-Hours support model - training manual** | Mother-infant dyads enrolled in the WIC breastfeeding peer counseling program  WIC staff and administrators (peer counselors, IBCLCs/nutritionists, BF coordinators, local and state WIC admins) | **Integrated comprehensive platform** that allows for: two-way text messaging; automated text messaging; 1-800 call center integration; video conferencing; regular reports for WIC admins  Peers to oversee and manage the platform  IBCLCs for peers to yield to  WIC local agency administrator support and buy-in  LATCH Community and Scientific Advisory Group (CSAG) | Training WIC staff and administrators on the **LATCH** and **Off-Hours** protocols  Administration of the automated text messaging schedule (message frequency depends on baby’s gestational age at enrollment) | Mother-infant dyads enrolled in the WIC breastfeeding peer counseling program | Evaluation of **LATCH** and **Off-Hours support model** trainings via survey and feedback forms  Weekly/monthly reports to WIC admins on **LATCH** platform use statistics  Weekly/monthly reports to WIC admins on **Off-Hours support** use statistics (number of calls, BF issue discussed and follow-up to resolve BF issues – closing the continuity of care loop) | Early contact between PC and mom at baby’s birth  Communication feedback loops between on-call **Off-Hours** team and local WIC agency offices for continuity of care | BF outcomes:  Exclusive, partial, and no BF  WIC postpartum nutrition package: full, partial, or no BF |

*LATCH model in green; off-hours PC support model in blue.
