# Supplementary material for: Evaluation and adaptation of a two-way text messaging intervention in the WIC breastfeeding peer counseling program: A qualitative analysis
Source: PLoS One. 2025 Jan 9;20(1):e0313779. doi: 10.1371/journal.pone.0313779 (PMC11717301; doi:10.1371/journal.pone.0313779)
Supplement: S2 Table — (DOCX) [file pone.0313779.s004.docx]

**Supplementary Materials**

**S2 Table. Mapping the Program Impact Pathways Framework to the Capability, Opportunity, Motivation and Behavior (COM-B) System for Characterizing and Designing Behavior Change Interventions***

The following definitions are used in the COM-B system (emphasis my own) [1]: **Capability** is defined as “the individual’s *psychological and physical capacity to engage* in the activity concerned.” Capability is further defined as physical and psychological capability, where the latter is defined as “the capacity to engage in the necessary thought processes – comprehension, reasoning, etc.” **Motivation** is defined as “all those *brain processes that energize and direct behavior*, not just goals and conscious decision-making” including “habitual processes, emotional responding, as well as analytical decision making.” Motivation is subdivided into “reflective processes (involving emotions and plans) and automatic processes (involving emotions and impulses that arise from associative learning and/or innate dispositions.” **Opportunity** is defined as “all the factors that *lie outside the individual* that make the behavior possible or prompt it.” Opportunity is divided into “physical opportunity afforded by the environment and social opportunity afforded by the cultural milieu that dictates the way we think about things.”

| **Component of the LATCH Model** | **COM-B Element** |
| --- | --- |
| LATCH Feasibility Trial Program Impact Pathways Framework in the WIC Program (2014-2016) | |
| LATCH text message protocol and platform training curriculum | Capability (psychological) |
| Train WIC peer, IBCLC, and other staff on the LATCH text messaging protocol and platform | Capability (psychological) |
| Outreach and recruitment by PCs to WIC mothers about LATCH (two-way text messaging) | Opportunity (physical) |
| Mothers exposed to the automated text messages on a regular schedule | Capability (psychological)  Motivation (reflective)  Opportunity (social) |
| Text message repetition and positive reinforcement from PC | Capability (psychological)  Motivation (reflective) |
| Mothers engage in two-way text message conversations with PC | Capability (psychological)  Motivation (reflective)  Opportunity (physical and social) |
| Mothers improve their BF knowledge, self-efficacy, and BF planning behaviors | Capability (psychological) |
| Mothers have the skills and self-efficacy to advocate for themselves in-hospital to receive BF support | Capability (psychological)  Motivation (reflective) |
| Mothers have improved communication with their PC (compared to those not enrolled in LATCH) | Capability (psychological)  Opportunity (physical and social) |
| Increased BF intensity and duration (compared to those not enrolled in LATCH) | Behavior |
| Adapted LATCH Program Impact Pathways Framework with Off-Hours PC Support in the WIC Program (future) | |
| LATCH text message protocol and platform training curriculum | Capability (psychological) |
| Train WIC peer, IBCLC, and other staff on the LATCH text messaging protocol and platform | Capability (psychological) |
| *Train WIC peer, IBCLC, and other staff on the LATCH off-hours protocol and platform* | Capability (psychological) |
| *Marketing****/***Outreach and recruitment by PCs to WIC mothers about LATCH (two-way text messaging) | Opportunity (physical) |
| *LATCH Marketing Strategy* | Opportunity (physical) |
| *LATCH off-hours protocol and platform training curriculum* | Capability (psychological) |
| Mothers exposed to the automated text messages on a regular schedule | Capability (psychological)  Motivation (reflective)  Opportunity (social) |
| *Mothers exposed to off-hours peer counseling support as needed* | Capability (psychological)  Motivation (reflective and automatic)  Opportunity (social) |
| *Use of translation services as needed* | Opportunity (physical) |
| Text message repetition and positive reinforcement from PC | Capability (psychological)  Motivation (reflective) |
| Mothers engage in two-way text message conversations with PC | Capability (psychological)  Motivation (reflective)  Opportunity (physical and social) |
| Mothers improve their BF knowledge, self-efficacy, and BF planning behaviors | Capability (psychological) |
| Mothers have the skills and self-efficacy to advocate for themselves in-hospital to receive BF support | Capability (psychological)  Motivation (reflective) |
| Mothers have improved communication with their PC (compared to those not enrolled in LATCH) | Capability (psychological)  Opportunity (physical and social) |
| Increased BF intensity and duration (compared to those not enrolled in LATCH) | Behavior |
| *Increase in the number of mothers enrolled in the Full BF WIC food package (compared to those not enrolled in LATCH)* | Behavior |

*Items in italics are suggested adaptations to the LATCH Program Impact Pathways Framework

References

[1] S. Michie, M. M. Van Stralen, and R. West, "The behaviour change wheel: a new method for characterising and designing behaviour change interventions," *Implement Sci,* vol. 6, pp. 1-12, 2011.
